# MISSION AND VISION OF THE MULTISITE VA ELIZABETH DOLE CENTER OF EXCELLENCE (EDCOE) FOR VETERAN AND CAREGIVER RESEARCH

**DOI:** 10.1093/geroni/igac059.170

**Published:** 2022-12-20

**Authors:** Ranak Trivedi

## Abstract

Enabling Veterans to remain safely in their homes is a Department of Veterans Affairs (VA) priority. Informal caregivers play a critical role in supporting Veterans with functional impairments through their own efforts and the use of home and community-based services (HCBS). The Elizabeth Dole Center of Excellence (EDCoE) for Veteran and Caregiver Research was created in 2018 as part of the VA Choose Home Initiative to expand VA capacity to deliver integrated, Veteran- and caregiver-partnered, data-driven approaches to care through a set of complimentary projects. The EDCoE is a virtual center, with its multi-disciplinary team spread across the country at 5 different VA Medical Centers. The team also collaborates with other VA caregiver researchers. Finally, the EDCoE is closely networked with national, regional, and local operational partners, allowing for rapid dissemination of findings. In this symposium, we will first discuss the mission and vision of the EDCoE. We will then discuss multiple projects that are led through the EDCoE. Paper 1 (San Antonio) will identify measurement gaps to guide monitoring and improving HCBS. Paper 2 (Miami/Canandaigua) will describe a national survey of 8000+ Veterans and 3500+ caregivers. Paper 3 (Palo Alto) will describe caregivers’ barriers to accessing VA and non-VA HCBS. Paper 4 (Salt Lake City/Canandaigua) will describe the results of latent trajectory analyses of administrative data to identify groups of Veterans who use different combinations of HCBS and institutional services. Our results are used by VA operational partners and policymakers to improve services for Veterans and informal caregivers.

